# Hyaluronic acid ameliorates the proliferative ability of human amniotic epithelial cells through activation of TGF-β/BMP signaling

**DOI:** 10.7717/peerj.10104

**Published:** 2020-09-30

**Authors:** Ya-Bing Tian, Nuo-Xin Wang, Yan Xu, Chang-Yin Yu, Ru-Ming Liu, Yi Luo, Jian-Hui Xiao

**Affiliations:** 1 Zunyi Municipal Key Laboratory of Medicinal Biotechnology, Affiliated Hospital of Zunyi Medical University, Zunyi, China; 2 Center for Translational Medicine, Affiliated Hospital of Zunyi Medical University, Zunyi, China; 3 Department of Neurology, Affiliated Hospital of Zunyi Medical University, Zunyi, China

**Keywords:** Hyaluronic acid, Human amniotic epithelial cells, Pro-proliferation, TGF-β/BMP signaling pathway

## Abstract

Human amniotic epithelial cells (hAECs) are a useful and noncontroversial source of stem cells for cell therapy and regenerative medicine, but their limited proliferative ability hinders the acquisition of adequate quantities of cells for clinical use due to not expressing telomerase in hAECs. Our previous study showed that hyaluronic acid (HA), an important component of the extracellular matrix, promoted the proliferation of human amniotic mesenchymal stem cells. Herein, we hypothesize that HA might improve the proliferative capability of hAECs. In the present study, the role of HA on the proliferation of human amniotic epithelial cells (hAECs) in vitro was investigated for the first time. HA at molecular weight of 300 kDa showed an obvious pro-proliferation effect on hAECs. Furthermore, HA not only kept phenotypic characteristics and differentiation capabilities of hAECs, but significantly promoted the secretion of the anti-inflammatory factors such as IL-10 and TGF-β1, and the expression of stem cell pluripotent factors such as Oct4 and Nanog. Analysis of PCR microarray data and RT-qPCR validation showed that TGF-β/BMP signaling was activated in the presence of HA. Further study showed that SB431542, an inhibitor of the TGF-β/BMP signaling, significantly suppressed the mRNA expression of *TGFBR3, BMP4, BMP7, BMPR1B, SMAD3, SMAD4*, and the pro-proliferative effect of HA on hAECs. These data suggest that HA is a safe and effective enhancer for in vitro expansion of hAECs, whose regulatory mechanism involves the TGF-β/BMP signaling.

## Introduction

Human amniotic epithelial cells (hAECs) emerge as a kind of novel stem-like cells isolated from the epithelium of the amnion (Silini et al., 2015). Since they are originated from an early embryological stage, hAECs retain the multipotency of early epiblasts, and can express the specific markers of human embryonic stem cells (ESCs) and differentiate into cells of all three germ layers (Miki et al., 2005; Lin et al., 2015). Furthermore, they can express biomarkers of multiple kinds of cells, such as epithelial cells, pancreatic cells, hepatocytes, and neural cells, suggesting their multifunctional potentials (Miki et al., 2005; Miki, 2018). They express quite low levels of the major histocompatibility complex (MHC) class I (MHC-I) molecules and do not express MHC II molecules on their surface, and thus have low immunogenicity (Miki et al., 2005). They can also secrete a variety of cytokines to modulate the immunoenvironment (Miki, 2018). Because hAECs are from the placenta tissue, which is commonly regarded as a medical waste after delivery, hAECs are easy and noninvasive to acquire, without the medically ethical and legal restrictions (Miki et al., 2005; Gramignoli et al., 2016). Unlike human ESCs, hAECs do not express telomerase and thus are not tumorigenic upon transplantation (Miki, 2018). Based on these excellent properties, hAECs have been widely applied in the pre-clinical treatment of many diseases, e.g., liver cirrhosis (Lin et al., 2015), pulmonary damages (Zhu et al., 2017), neurological disorders (Sankar & Muthusamy, 2003), autoimmune uveitis (Li et al., 2018), diabetes (Luo et al., 2019), early Achilles tendon defects (Barboni et al., 2018), as well as aging-related diseases (Di Germanio et al., 2016). Therefore, hAECs possess considerable therapeutic potentials in regenerative medicine.

Like many other stem cells, however, it is a major challenge to obtain enough clinical-grade hAECs in appropriate conditions due to the limited number of hAECs from a single donor, and the limited proliferative ability owing to negative expression of telomerase in hAECs (Motedayyen et al., 2017). Although transfection of the genes of human telomerase reverse transcriptase and origin-defective simian virus 40 large T-antigen into hAECs greatly elevated the cellular proliferative capability of hAECs (Zhou et al., 2013), the gene instability and tumorgenicity of these genetically modified cells are great hurdles for future therapeutic applications. Thus, a safe and effective approach to expand hAECs in vitro needs to be established. Recent publications indicated that addition of bioactive substrates into culture medium is a possible strategy. For example, some growth factors, such as epidermal growth factor (EGF), have been illustrated to promote the proliferation of hAECs through cell cycle regulation (Fatimah et al., 2012; Ochsenbein-Kölble et al., 2003). Nonetheless, addition of EGF caused side effects such as decreasing the expression of stem cell pluripotent factors (*Oct-3/4*, *Sox2*, and *Nanog*) and other cell biomarkers (*NSE*, *NF-M*, and *MAP2*) (Fatimah et al., 2012). Therefore, other additives that could promote the proliferation of hAECs are worth exploring.

Hyaluronic acid (HA), an important component of the extracellular matrix in the human body, has excellent biocompatibility and diverse physiological functions, and has been employed as a multifaceted regulator for cell activities, such as cellular division, adhesion, migration and differentiation (Fakhari & Berkland, 2013; Dicker et al., 2014). Previous studies highlighted the pro-proliferative effects of HA on several kinds of cells in vitro. For example, 900 kDa HA could promote the proliferation of patient-derived meniscus cells potentially via PI3K/MAPK signaling pathway (Murakami et al., 2019); oligosaccharide HA (oHA, 4–20 mer, ∼1.6–8.0 kDa) could stimulate the proliferation of human umbilical vein endothelial cells (hUVECs) by oHA-ezrin signaling axis (Mo et al., 2011); our previous study indicated that 300 kDa HA could accelerate the proliferation of human amniotic mesenchymal stem cells (hAMSCs) through the Wnt/β-catenin signaling pathway (Liu et al., 2016). Nonetheless, effects of HA on the proliferative ability of hAECs remain unanswered.

In the present study, we found that 300 kDa HA could ameliorate the proliferative ability of hAECs through the activation of TGF-β/BMP signaling. What’s more, the secretion of multipotency factors and anti-inflammatory cytokines in hAECs was increased and the multipotent differentiation ability of hAECs was well maintained.

## Materials & Methods

### Reagents

Dulbecco’s modified Eagle medium with low glucose (LG-DMEM), F-12 medium, trypsin, GlutaMAX, non-essential amino acids, fetal bovine serum (FBS), and adipocyte/osteocyte/chondrocyte differentiation basal medium and their corresponding supplements were purchased from Gibco (NY, USA). Antibiotics (penicillin and streptomycin) were purchased from Sino American Biotechnology (Shanghai, China). HA was obtained from Seebio Biotech, Inc. (Shanghai, China). Bovine serum albumin (BSA), hematoxylin, saturated oil red O dye solution, Marker I DNA Ladder, and Gold View Type I nucleic acid staining were obtained from Solarbio (Beijing, China). Alizarin red dye solution and Toluidine blue were purchased from Cyagen (Shanghai, China) and Sangon Biotech (Shanghai, China), respectively. SB431542 was obtained from Selleck (Shanghai, China). Cell Counting Kit-8 (CCK-8) was purchased from Dojindo (Beijing, China). Human IL-10 ELISA Kit and human TGF-β1 ELISA Kit were purchased from Biosamite (Shanghai, China). All other chemicals were of analytical grade.

### Ethics

All amniotic membranes were collected from healthy donors undergoing caesarean delivery and the informed written consent was acquired from the donor prior to tissue collection. The study protocol and the use of the human amniotic membrane were approved by the Animal Experiment Ethics Committee of Zunyi Medical University (Zunyi, China) (License number granted: (2014)2-085) and Medical Ethics Committee of Zunyi Medical University (License number granted: (2014)1-042) and the declaration of Helsinki was strictly abode by the investigators.

### Isolation and cultivation of hAECs

hAECs were obtained by mechanical separation and the following enzymatic digestion. Briefly, the amniotic membranes were mechanically separated from the chorion of fresh term placentae. After completely washing away the blood with D-Hank’s solution containing penicillin (100 U/mL) and streptomycin (100 µg/mL), the amnions were minced and transferred into a 50-mL tube containing 0.05% trypsin and 0.02% EDTA solution. Then, the tube was maintained at 37 °C for 40 min in a water bath shaker at 180 rpm. The digested tissue pieces were filtered through a 300 mesh stainless steel sieve. The cell suspension was collected by centrifugation and resuspended in LG-DMEM/F12 complete medium (containing 10% FBS) to obtain primary hAECs (passage 0, P0). Cells were inoculated into T25 flasks at a density of 2. 5 ×10^6^/flask and were cultured at 37 °C in a 5% CO_2_ incubator, and the medium was replaced once every 72 h. Cells were passaged when they reached 80% confluence. All of the following assays repeated at least for 3 times and each experimental replicate used a batch of hAECs derived from a different placental donor. Cells from multiple batches were not mixed together and cultured.

### Phenotypic properties of hAECs

The phenotype of hAECs was validated by flow cytometric analysis in Affiliated Hospital of Zunyi Medical University. Cells were incubated with antibodies (mouse anti human, BD, NJ, USA) against CD44 (Cat# 555478) and CD45 (Cat# 561865), conjugated to fluorescein isothiocyanate, antibodies against CD34 (Cat# 550619), CD29 (Cat# 557332), CD73 (Cat# 550257), CD166 (Cat# 559263), conjugated to phycoerythrin, and the antibody against HLA-DR (Cat# 552764) conjugated to PerCP-Cy5.5, at room temperature for 25 min in dark, and analyzed by a FACSCalibur system (BD, Franklin Lakes, NJ, USA) using CellQuest software after PBS washing. Non-immune isotype IgG antibodies (BD, NJ, USA) were used as controls. Similar to our previous study (Luo et al., 2019), hAECs highly expressed the surface markers such as CD29 (99.62%), CD73 (99.60%), and CD166 (99.39%), and lowly expressed CD44 (17.6%), but did not express the hematopoietic stem cells marker CD34 (0.09%) and CD45 (1.17%), and the MHC II molecule HLA-DR (0.02%). The absence of CD34 and CD45 positive cells indicated that the isolates were not contaminated with hematopoietic stem cells from umbilical cord blood or embryonic fibroblasts.

### Immunocytochemistry

The first generation (passage 1, P1) hAECs in logarithmic growth phase were seeded in 6-well plates at a density of 3. 0 ×10^5^/well, and cytokeratin 19 (CK19) and vimentin were measured by immunocytochemical staining. In brief, cells were rinsed with D-PBS and fixed in 4% paraformaldehyde for 30 min at 24−28 °C. Cells were then treated with 0.5% Triton-X 100 for 10 min and 5% BSA for 30 min at 24−28 °C, followed by incubation with antibodies (mouse anti human, 1:100, Gene Tech, Chengdu, China) against CK19 (Cat# GM088804) or vimentin (Cat# GM072504) overnight at 4 °C. Cells were then washed and incubated with horseradish peroxidase (HRP)-conjugated goat anti-mouse IgG secondary antibody for 30 min at 37 °C. Finally, the labeled cells were stained using a DAB kit (Gene Tech, Chengdu, China) and observed under a microscope after washing. Nuclei were counterstained with hematoxylin. Cells incubated with PBS were used as controls.

### Proliferation assay

The P1 hAECs in the logarithmic growth phase were seeded in 96-well plates at densities of 1, 2, 4, 8, 16, 32 and 64 ×10^3^/well, respectively. After incubation at 37 °C for 6 h, cells were treated with CCK-8 kit according to the manufacturer’s instruction. The absorbance of the cells in each well was measured at 450 nm using a microplate reader (MultiskanTM GO, ThermoFisher, USA), and a standard curve was subsequently generated and used to calculate the cell numbers. To explore the effect of HA with different molecular weights on cell proliferation, the P1 hAECs in logarithmic growth phase were seeded in a 96-well plate at a density of 1. 0 ×10^4^/well, the medium was replaced on day 3 after inoculation. The cells were then treated for 48 h by 0.5 mg/mL of HA with different molecular weights including 50 kDa, 300 kDa, and 1,000 kDa. Finally, the proliferation capability was measured by CCK-8 assay following the manufacturer’s instructions. To evaluate the effect of 300 kDa HA at different doses on cell proliferation, the P1 hAECs in logarithmic growth phase were seeded in a 96-well plate at a density of 1. 0 ×10^4^ cells/well, the medium was replaced on the day 3 after inoculation. The cells were then treated for 48 h with 300 kDa HA at different doses of 0.05, 0.1, 0.5, and 1 mg/mL. The proliferation capability was measured by CCK-8 assay. Cell doubling time (DT) was calculated by the following formula (Zhou et al., 2013): DT = t × log2/(logN_t_−logN_0_), where t was treatment time with HA, N_0_ was the seeded cell number, N_t_ was the harvested cell number after HA treatment for t hours. In this case, t was 48 h.

### in vitro differentiation potentials

P1 hAECs in logarithmic growth phase were inoculated into 6-well plates at a density of 3. 6 ×10^5^/well. hAECs in the presence and absence of HA (300 kDa, 1 mg/mL) were incubated in LG-DMEM/F12 complete medium at 37 °C for 3 days, and the medium was subsequently replaced with differentiation complete media for adipocyte, osteocyte, and chondrocyte, respectively. Adipogenic differentiation complete medium was composed of LG-DMEM supplemented with Dex (1 µM), indomethacin (200 µM), IBMX (500 µM), and recombinant human insulin (20 mg/mL). Osteogenic differentiation complete medium was composed of LG-DMEM supplemented with Dex (0.1 µM), β-glycerol phosphate (5 mM) and ascorbic acid 2-phosphate (50 µg/mL). Chondrogenic differentiation complete medium was composed of LG-DMEM supplemented with Dex (0.1 µM), ascorbic acid 2-phosphate (50 µg/mL), TGF-β3 (10 ng/mL) and 1% ITS. These differentiation media were replaced every 3 days. After induction, differentiation properties were assayed on the 14th day for adipogenesis, and on the 21st day for osteogenesis and chondrogenesis. Briefly, cells were fixed using 4% paraformaldehyde for 30 min and washed with PBS before staining. For adipogenic staining, cells were stained with oil red O staining solution (saturated oil red O solution: ddH_2_O = 3:2, then filtered with neutral filter paper). For osteogenic staining, cells were stained by Alizarin red. For chondrogenic staining, cells were stained with Toluidine blue solution.

### Immunofluorescence assay

P1 hAECs in logarithmic growth phase were inoculated into 6-well plates at a density of 3 ×10^5^/well, and cultured at 37 °C with 5% CO_2_ for three days in LG-DMEM/F12 complete medium. The cells were further cultured in LG-DMEM/F12 complete medium with or without HA (300 kDa, 1 mg/mL) for two days. Immunofluorescence staining was then performed to detect the expression of stemness-related proteins (Nanog, Sox-2 and Oct-4). Briefly, after fixation, washing, and blocking steps, as did in the immunocytochemistry assay, the cells were incubated with rabbit-derived primary antibodies (Abcam, Cambridge, UK) against Nanog (1:200; Cat# ab109250), Sox-2 (1:100; Cat# ab137385), and Oct-4 (1:250; Cat# ab200834) at 4 °C overnight. The cells were then washed by PBS thrice and incubated with a FITC-conjugated goat anti-rabbit IgG secondary antibody (1:32, Abcam, Cambridge, UK) for 2 h at 24−28 °C. The cells were washed thrice again to remove the secondary antibody and then counterstained with 4′,6-diamidino-2-phenylindole (DAPI, Roche, Basel, Switzerland). Cells were then observed under a fluorescent microscope (Leica DMIRB, DIC, Germany). Goat serum was used as a negative control.

### Enzyme-linked immunosorbent assay

Enzyme-linked immunosorbent assay (ELISA) was performed according to the manufacturer’s instructions. After the cell supernatants of each group were collected at 24−28 °C, the target proteins were concentrated by centrifugation for 10 min at 4,000 g. The supernatant of cells was concentrated to 1/12 of its original volume. The ELISA kit was equilibrated at 24−28 °C for 20 min, followed by the addition of 50 µL of different concentrations of standards to each standard well, and 10 µL of the sample to be tested and 40 µL of the sample diluents to each testing well. One hundred microliters of horseradish peroxidase (HRP)-labeled detection antibody were added to each standard well and sample well. Then the reaction well was sealed and incubated at 37 °C for 60 min. After repeated washing, substrates A and B (each 50 µL) were successively added to each well for color development. The samples were incubated at 37 °C in the dark for 15 min. Finally, 50 µL of stop solution was added to each well and the OD value was measured at 450 nm within 15 min using a platereader (MultiskanTM GO, ThermoFisher, USA).

### Cellular senescence assay

To evaluate the effect of HA on cellular senescence, P1 hAECs in logarithmic growth phase were inoculated into 6-well plates at a density of 3 ×10^5^/well and P2 hAECs were cultured in the presence or absence of HA (300 kDa, 1 mg/mL) for 48 h. After cell passage, cellular senescence of P3 hAECs was detected using β-Galactosidase Staining Kit (Beyotime, Shanghai, China) following the manufacturer’s instructions. The stained cells were then observed under a microscope and cells from 9 random fields were selected to count the rate of SA-β-Gal positive cells to total cells for each group.

### RT-qPCR analysis

RNA was extracted from all samples using the RNAiso Plus Kit (TaKaRa, Dalian, China) according to the manufacturer’s protocol. The concentration and purity of extracted RNA were measured using the NanoDrop 2000c (Thermo Scientific, Bonn, Germany) at OD260 and OD260/280, respectively. Reverse transcription was conducted using the PrimeScript™ RT reagent Kit (TaKaRa, Dalian, China) according to the manufacturer’s instructions. PCR was carried out using the Premix Ex TaqTM II (for Real Time) (TaKaRa, Dalian, China) on a CFX96 Touch real-time PCR detection system (Bio-Rad, Hercules, CA, USA). The primers used in the reaction are shown in Table 1. The 2^−ΔΔCt^ method was used to quantify gene expression levels. β-Actin was used as the internal control for normalization.

**Table 1:** Primer sequences of target genes.

Gene	Sequence (5′→3′)	Genbank ID	Length of product (bp)
*Nanog*	For: CCCCAGCCTTTACTCTTCCTA Rev: CCAGGTTGAATTGTTCCAGGTC	NM_024865.3	97
*Sox-2*	For: TACAGCATGTCCTACTCGCAG Rev: GAGGAAGAGGTAACCACAGGG	NM_003106.3	110
*Oct-4*	For: AAGGATGTGGTCCGAGTGTG Rev: GAAGTGAGGGCTCCCATAGC	NM_002701.5 NM_203289.5	180
		NM_001173531.2	
		NM_001285986.1	
		NM_001285987.1	
*Ki67*	For: GAAGAGGTCCTACCAGTCGGC Rev: CCTCTCCATCCCAGTTCCATAG	NM_00114596.1	156
*PCNA*	For: GACTCGTCCCACGTCTCTTTGG	NM_002592.2	168
	Rev: CGCGTTATCTTCGGCCCTTA	NM_182649.1	
*BMP3*	For: CCCCAGCCTTTACTCTTCCTA Rev: CCAGGTTGAATTGTTCCAGGTC	NM_ 024865.3	97
*BMP4*	For: TACAGCATGTCCTACTCGCAG Rev: GAGGAAGAGGTAACCACAGGG	NM_003106.3	110
*BMP6*	For: AGCGACACCACAAAGAGTTCA Rev: GCTGATGCTCCTGTAAGACTTGA	NM_001718.5	159
*BMP7*	For: AAGGATGTGGTCCGAGTGTG Rev: GAAGTGAGGGCTCCCATAGC	NM_002701.5 NM_203289.5	180
*TGFBR3*	For: TACAGCATGTCCTACTCGCAG Rev: GAGGAAGAGGTAACCACAGGG	NM_003106.3	110
*BMPR1B*	For: AAGGATGTGGTCCGAGTGTG Rev: GAAGTGAGGGCTCCCATAGC	NM_002701.5 NM_203289.5	180
*SMAD2*	For: CCGACACACCGAGATCCTAAC Rev: GAGGTGGCGTTTCTGGAATATAA	NM_005901.5 NM_001135937.2	125
		NM_001003652.3	
*SMAD3*	For: TGGACGCAGGTTCTCCAAAC Rev:CCGGCTCGCAGTAGGTAAC	NM_005902.3 NM_001145102.1	90
		NM_001145103.1	
		NM_001145104.1	
*SMAD4*	For: CTCATGTGATCTATGCCCGTC Rev: GAGGAAGAGGTAACCACAGGG	NM_003106.3	110
*SMAD5*	For: CCAGCAGTAAAGCGATTGTTGG Rev: GGGGTAAGCCTTTTCTGTGAG	NM_005903.6 NM_001001419.2	220
*β-actin*	For: TGGCACCCAGCACAATGAA Rev:CTAAGTCATAGTCCGCCTAGAAGCA	NM_001001420.2	186

### PCR microarray

The potential signaling pathways by which HA affected the proliferation of hAECs was preliminarily screened by a PCR microarray. P1 hAECs in logarithmic growth phase were harvested, inoculated into 6-well plates at 3 ×10^5^/well, and cultured with 5% CO_2_ at 37 °C. The medium was changed after 3 days, and then the cells were cultured for 18 or 36 h in the presence and absence of HA (300 kDa, 1 mg/mL). The total RNA was purified using the RNeasy MinElute kit (Qiagen, Valencia, CA, USA) according to the manufacturer’s instructions. cDNA was synthesized using a SuperScript III Reverse Transcriptase Kit (Invitrogen, Shanghai, China). Real-time PCR was conducted, and the 2^−ΔΔCt^ method was used to quantify gene expression levels. The signal was collected by Agilent Feature Extraction software and analyzed by Agilent GeneSpring GX software.

### Validation of the signaling pathway

P1 hAECs in logarithmic growth phase were harvested, inoculated into 6-well plates at 3 ×10^5^/well, and cultured with 5% CO_2_ at 37 °C. The medium was changed after 3 days, and the cells were then treated for 48 h in the presence or absence of HA (300 kDa, 1 mg/mL). The expression of differential genes related to the TGF-β/BMP signaling pathway revealed by the PCR microarray was validated using RT-PCR. To further unravel the direct correlation between the HA-induced cell proliferation and the TGF-β/BMP signaling pathway, we added SB431542 (10 µM), a specific blocker of the TGF-β/BMP signaling pathway, to the HA (300 kDa, 1 mg/mL)-treated group. Cell proliferation reflected by OD values were measured using the CCK-8 kit after 48 h of treatment. At the same time, the proliferation-related and TGF-β/BMP signaling pathway-related genes were detected by RT-PCR. Expression levels of proteins Smad2/3 and p-Smad2/3 were measured by Western Blot. The primary antibodies used were as follows: rabbit anti-human Smad2/3 (1:1000; Cat# 8828S, Cell Signaling Technology, Boston, USA), rabbit anti-human p-Smad2/3 (1:1000; Cat# 3102S, Cell Signaling Technology, Boston, USA), and rabbit anti-human GADPH (used as the reference protein) (1:1000; Cat# 10494-1-AP, ProteinTech Group, Chicago, USA). The secondary antibody used was HRP-labeled goat anti-rabbit IgG (1:3000; Cat# SA00001-2, ProteinTech Group, Chicago, USA). The protein bands were developed by ECL using Bio-Rad ChemiDocTM MP Imaging System, exposed using Image Lab software, and semi-quantified by ImagingJ software. The other three groups, including the negative control group treated without neither HA nor SB431542, the group treated with HA (300 kDa, 1 mg/mL) but without SB431542, and the group treated with SB431542 (10 µM) but without HA, were used as controls.

### Cell cycle assay

P1 hAECs in logarithmic growth phase were plated into T25 culture flasks at 7. 81 ×10^5^/flask, and cultured in 5% CO_2_ at 37 °C. The medium was changed after 3 days. Then, 300 kDa HA at different concentrations (0, 0.05, 0.1, 0.5, and 1 mg/mL) was added into the cells. After 48 h of treatment, the resultant cells were collected and stained with propidium iodide (PI) working solution according to the instruction of the Cell Cycle Kit (Beyotime, Shanghai, China). RNaseA had been included in the Kit to degrade RNA to ensure that only DNA was bound. The cell cycle was detected by flow cytometry (FACSCalibur, BD, Franklin Lakes, NJ, USA).

### Statistical analysis

All experimental data were expressed as mean ± standard deviation (sd) and statistically analyzed using ANOVA test and Student’s *t* test by SPSS 19.0 software. The normal distribution of the data is verified by Agostino-Pearson omnibus normality test. Post-hoc comparisons were performed using Tukey’s multiple comparisons test. *P* <  0.05 was considered to be statistically significant.

## Results

### Effects of HA on the proliferation of hAECs

**Figure 1:**
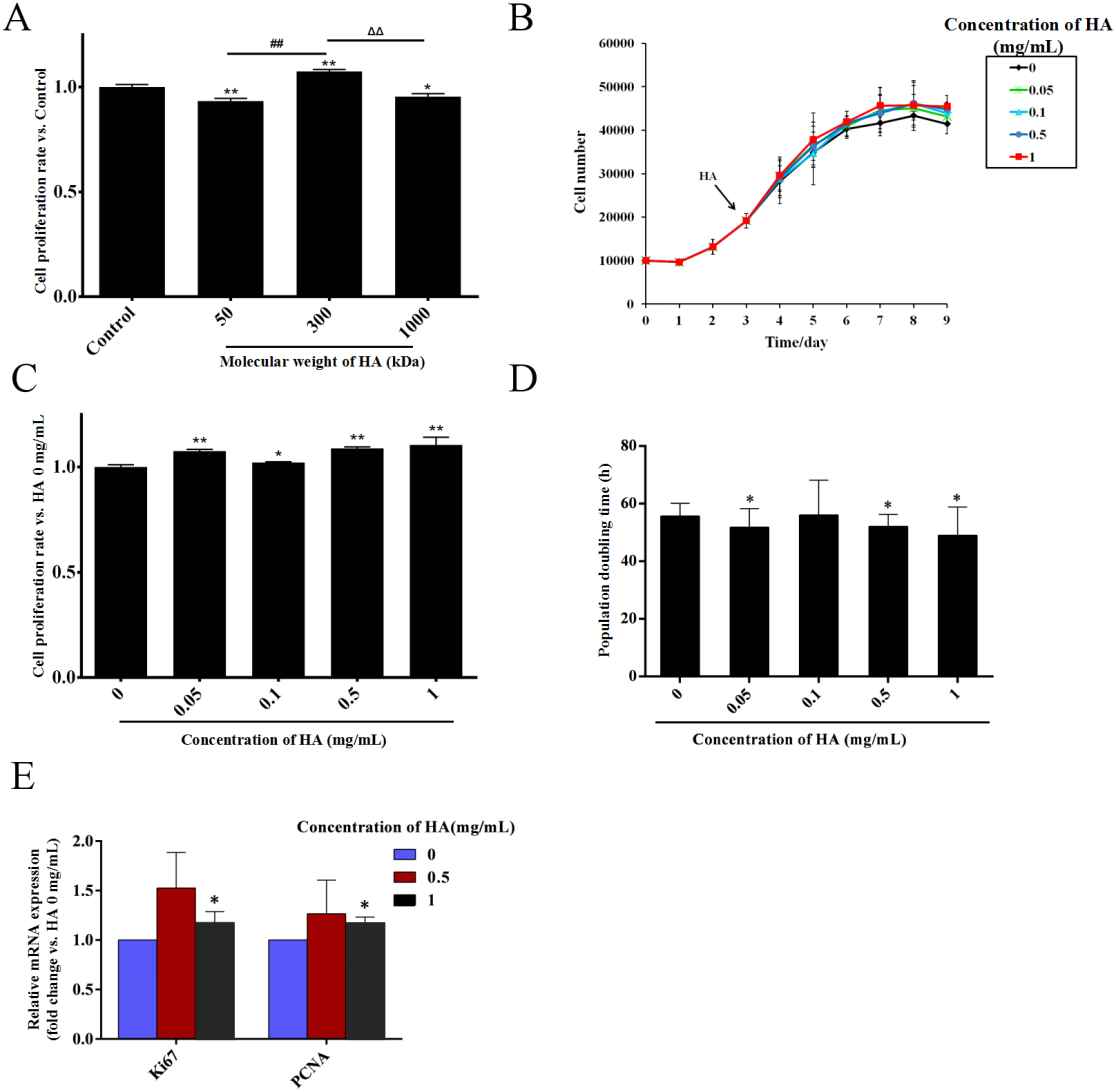
Effect of HA on the proliferation of hAECs. (A) Effect of HA molecular weight on the proliferation of hAECs (HA 50 kDa, 300 kDa, and 1,000 kDa; 0.5 mg/mL). Cell proliferation rate was normalized by the average value of the control. ^∗^*P* < 0.05, ^∗∗^*P* < 0.01 vs. control; ^##^*P* < 0.01 vs. 50 kDa HA group; ΔΔ*P* < 0.01 vs. 1,000 kDa HA group. (B) Effect of 300 kDa HA on the growth curve of hAECs. HA was added on the 3rd day as indicated by the arrow head. (C) Effect of 300 kDa HA on the proliferation of hAECs. Cell proliferation rate was normalized by the average value of the 0 mg/mL HA group. ^∗^*P* < 0.05, ^∗∗^*P* < 0.01 vs. control. (D) Effect of 300 kDa HA on the population doubling time (DT) of hAECs. ^∗^*P* < 0.05 vs. control. (E) Effect of 300 kDa HA on the expression of proliferation-related genes *Ki67* and *PCNA* of hAECs. ^∗^*P* < 0.05 vs. control. Scale bars: 50 µm. All data are expressed as mean ± sd (*n* =3). Cell number, DT, and gene expression level were all calculated after 48 h of HA addition.

HA is a kind of polysaccharide whose biological effects are greatly affected by its molecular weight (Cyphert, Trempus & Garantziotis, 2015). Thus, we firstly explored the effect of the molecular weight of HA on the proliferation of hAECs. The cells were exposed to 0.5 mg/mL HA with the molecular weights of 50, 300, and 1,000 kDa. After 48 h of treatment, compared with the control group without HA, the cell number in the 1,000 kDa and 50 kDa HA groups was decreased by 6.7% (*P* <  0.01) and 4.5% (*P* <  0.05), respectively, while the cell number in the 300 kDa HA group was increased by 7.4% (*P* <  0.01). These results indicate that 50 kDa and 1,000 kDa HA inhibited, yet 300 kDa HA promoted the proliferation of hAECs (Fig. 1A). The effect of 300 kDa HA at different concentrations on the proliferation of hAECs was further investigated (Fig. 1B). After 48 h of treatment, compared with the control group, the number of cells in the 0.05, 0.1, 0.5, and 1 mg/mL HA groups was increased by 7.6% (*P* <  0.01), 2.1% (*P* <  0.05), 8.8% (*P* <  0.01), and 10.5% (*P* <  0.01), respectively (Fig. 1C). Consistently, further study showed that the population doubling time (DT) of the cells was shortened from 58.47 h in the control group to 51.83 (*P* <  0.05), 56.37, 50.93 (*P* <  0.05) and 49.97 h (*P* <  0.05) in the 0.05, 0.1, 0.5, and 1 mg/mL HA groups, respectively (Fig. 1D). It is evident that 300 kDa HA could promote the proliferation of hAECs in the concentration range of 0.05–1 mg/mL, and the pro-proliferative effect of HA at 1 mg/mL was the most significant. We further measured the effect of 300 kDa HA on the expression of proliferation-associated genes *Ki67* and *PCNA* after 48 h of treatment. HA at 0.5 and 1 mg/mL could boost the transcription of *Ki67* and *PCNA* in hAECs, and in particular, HA at 1 mg/mL presented a significant increase as compared to the control group (*P* <  0.05) (Fig. 1E).

### Effects of HA on the morphological and phenotypic characteristics of hAECs

**Figure 2:**
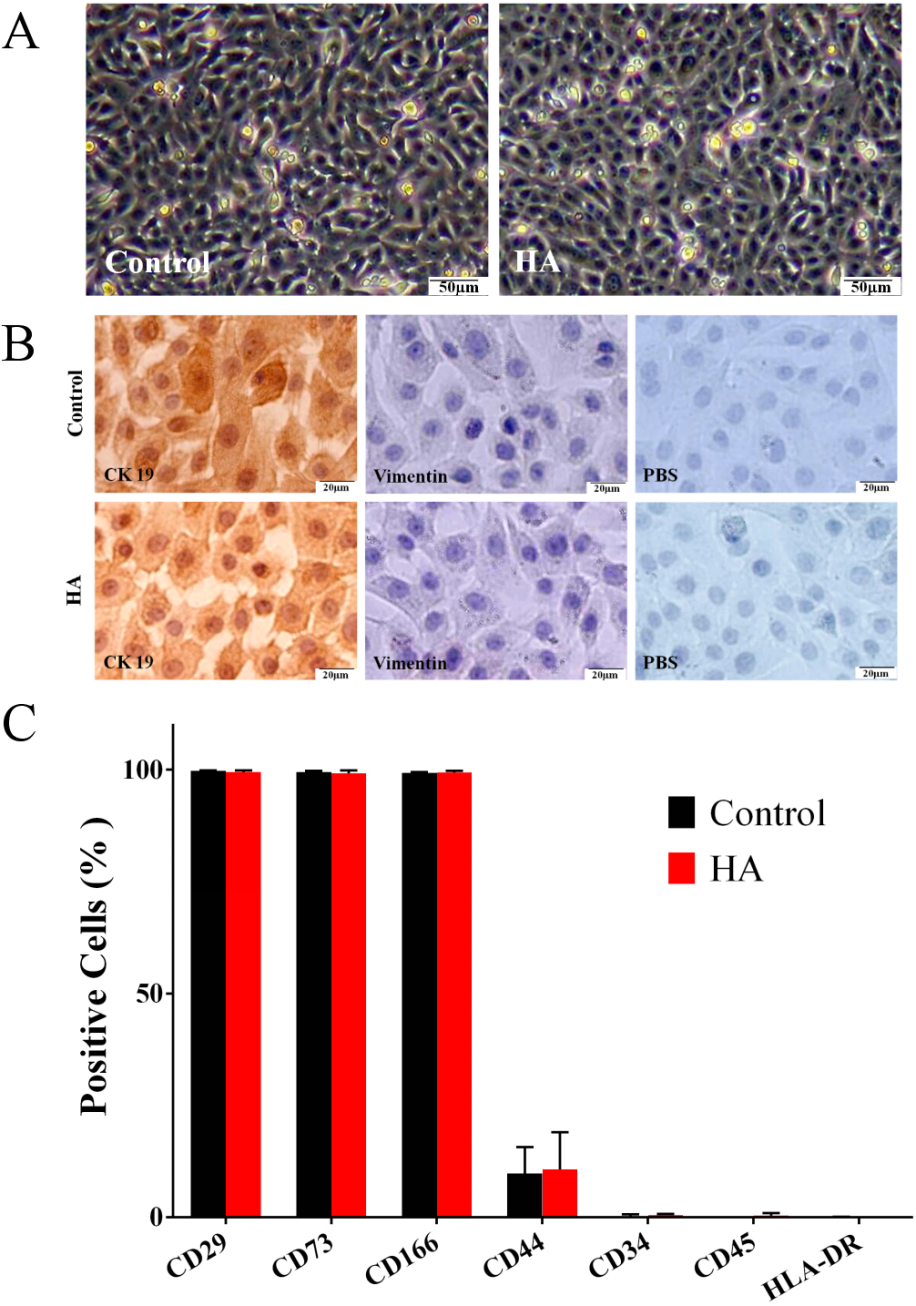
Effect of 300 kDa HA on morphological and phenotypic characteristics of hAECs. (A) Morphological characteristics. (B) Cytokeratin 19 (CK19) and vimentin expression. (C) Surface markers. The HA (300 kDa, 1 mg/mL) treatment time was 48 h. (% Positive) indicates the proportion of cells positively labeled by corresponding surface marker antibodies.

As shown in Fig. 2A, the morphology of hAECs after 48 h of HA (300 kDa, 1 mg/mL) treatment was consistent with that of the control group, exhibiting ovoid or triangular shapes with a typical paving stone-like arrangement. Immunocytochemistry staining indicates that after HA exposure, hAECs still expressed the epithelial cell marker cytokeratin 19 (CK19), but not the mesenchymal cell marker vimentin (Fig. 2B). Flow cytometric analysis shows that after HA treatment, hAECs still expressed high levels of CD29, CD73 and CD166, low level of CD44, and did not express the hematopoietic stem cell surface markers such as CD34, CD45, and HLA-DR (Fig. 2C and Fig. S1). The above results indicate that 300 kDa HA treatment does not alter the morphological and phenotypic characteristics of hAECs.

### Effects of HA on the stemness property and differentiation potential of hAECs

**Figure 3:**
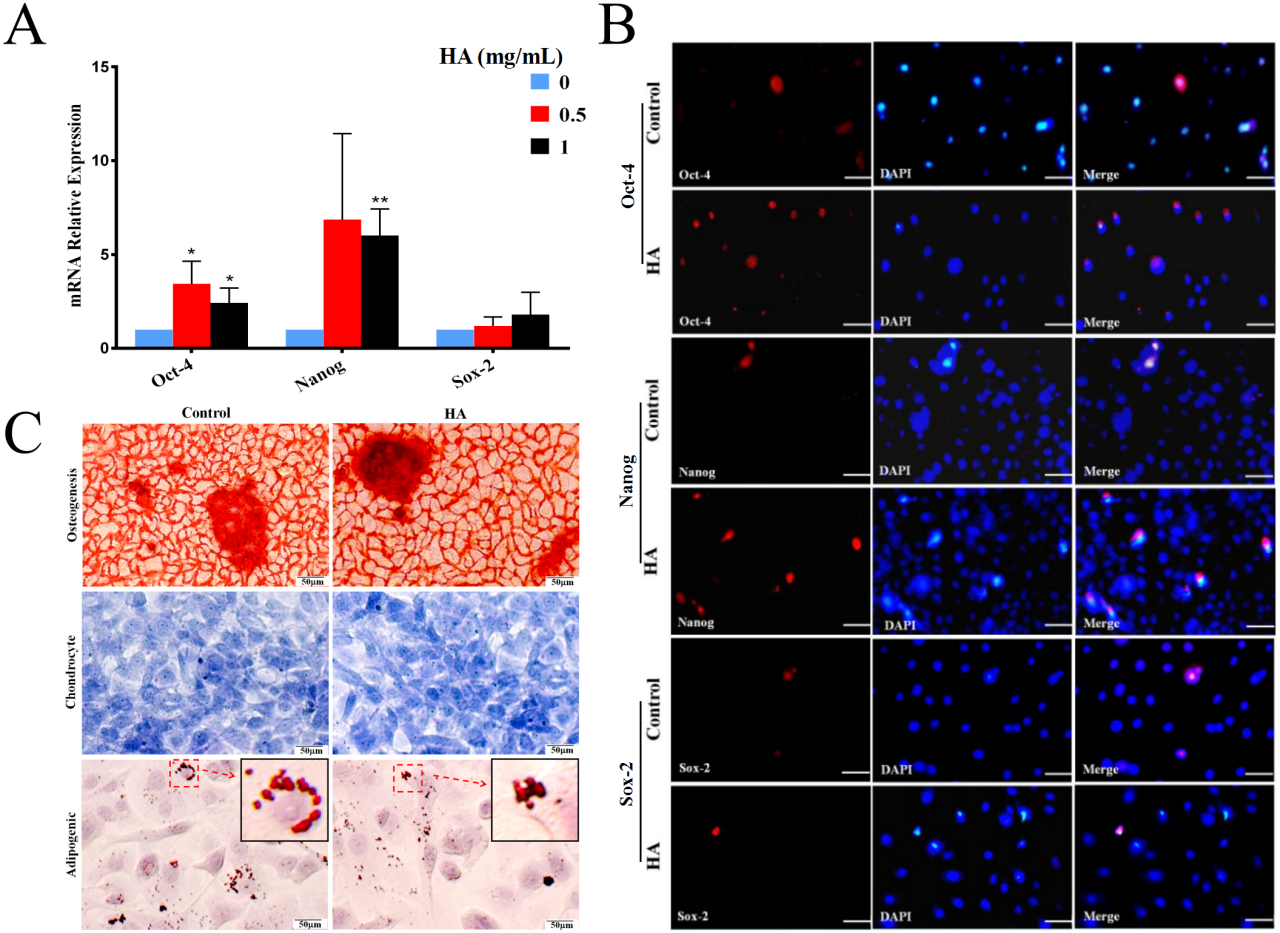
Effect of 300 kDa HA on the expression of pluripotency markers and the differentiation of hAECs. (A) Effect of HA on the expression of pluripotency-related genes. Gene expression was tested after 48 h of HA addition. The data are expressed as mean ± sd (*n* = 3), ^∗^*P* < 0.05, ^∗∗^*P* < 0.01 vs. control. (B) Effect of HA on the expression of pluripotency-related proteins. Protein staining was performed after 48 h of HA addition. Oct-4 (red), Nanog (red), and Sox-2 (red). Scale bars: 50 µm. (C) Effect of HA on the differentiation of hAECs into osteoblasts, chondrocytes and adipocytes. The hAECs were cultured in HA-containing medium for 3 days before induction by corresponding differentiation medium. The nuclei were stained with DAPI (blue). Scale bars: 50 µm. HA, 300 kDa, 1 mg/mL.

In comparison with the control, HA at 1 mg/mL significantly promoted the transcriptional levels (*P* <  0.01) of *Oct-4* and *Nanog* in hAECs, but only gave rise to a slight increase in *Sox-2* expression without significance (Fig. 3A). In agreement with the gene expression, levels of proteins Nanog and Oct-4 were markedly increased after 1 mg/mL HA treatment compared to the control group without HA (Fig. 3B). The result indicates that HA may act to enhance the maintenance of stemness in culture.

We also analyzed the multilineage differentiation capabilities of hAECs after HA treatment. Chemical staining reveals that, after induction, similar to the control, hAECs with HA treatment still generated bright red calcium salt nodules (osteogenic differentiation), purple-blue glycosaminoglycans (chondrogenic differentiation), and red lipid droplets (adipogenic differentiation), respectively (Fig. 3C). These results suggest that the multilineage differentiation capability of hAECs is well retained after HA treatment.

### Effects of HA on the paracrine action of hAECs

**Figure 4:**
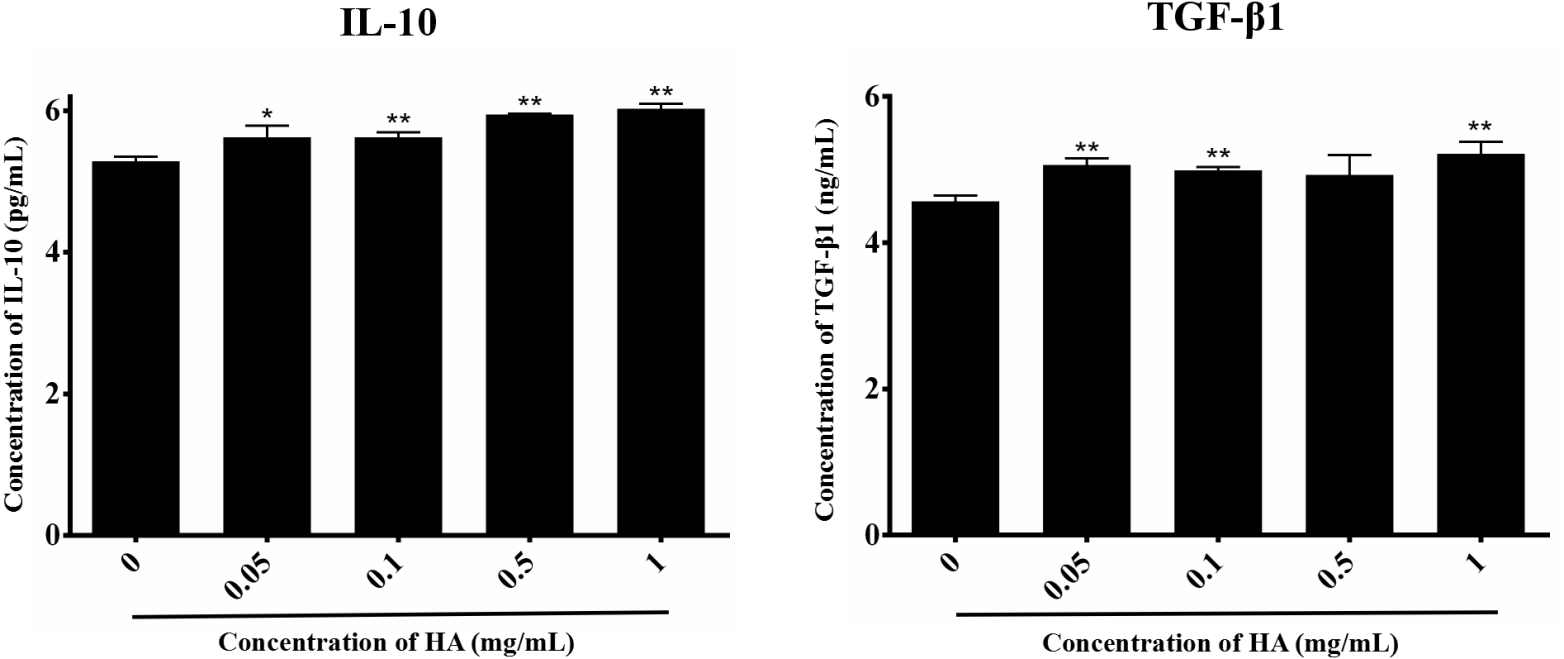
Effect of 300 kDa HA on anti-inflammatory cytokine secretion of hAECs. ^∗^*P* < 0.05, ^∗∗^*P* < 0.01 vs. control.

We then explored the effect of 300 kDa HA on the paracrine of two anti-inflammatory factors secreted by hAECs, IL-10 and TGF-β1 (Skeen et al., 2012), as shown in Fig. 4. After treatment with HA at different concentrations (0.05, 0.1, 0.5, and 1 mg/mL), IL-10 secretion for all groups was significantly increased when compared with the control group (*P* <  0.05 or *P* <  0.01). HA at the doses of 0.05, 0.1, and 1 mg/mL also significantly up-regulated the secretion of TGF-β1 when compared with the control group (*P* <  0.05). We could find that the secretion of IL-10 and TGF-β1 peaked at 6.03 ± 0.07 pg/mL and 5.22 ± 0.17 ng/mL, respectively, in the presence of 1 mg/mL HA. These results indicate that 300 kDa HA may enhance the secretion of anti-inflammatory cytokines in hAECs.

### Effects of HA on the cell cycle of hAECs

We also evaluated the effect of 300 kDa HA on the cell cycle of hAECs (Table 2 and Fig. S2). As HA dosage increased, the cell percentage in G0/G1 phase decreased from 88.38 ± 1.18% (0 mg/mL) to 79.69 ± 1.52% (1 mg/mL), while the cell percentage in S phase sharply elevated from 5.84 ± 0.24 (0 mg/mL) to 13.20 ± 1.87 (1 mg/mL) (with an unexpected reduction to 1.20 ± 1.32% at 0.05 mg/mL) (Table 2). Simultaneously, the cell percentage in the G2/M phase rose after HA treatment, but not in a dose-dependent manner. Collectively, we find that as the increase of 300 kDa HA doses, the percentage of cells in S and G2/M phases increase, indicating that 300 kDa HA could regulate the cell cycle transition and drive the cells into a proliferating state.

**Table 2:** Effect of 300 kDa HA on the cell cycle of hAECs.

HA concentration (mg/mL)	G_0_/G_1_ (%)	S (%)	G_2_/M (%)
0	88.38 ± 1.18	5.84 ± 0.24	5.78 ± 0.95
0.05	83.99 ± 0.74^**^	1.20 ± 1.32^**^	14.81 ± 1.80^**^
0.1	85.44 ± 1.24^*^	7.18 ± 0.66^*^	7.38 ± 1.11
0.5	82.15 ± 0.95^**^	8.22 ± 0.27^**^	9.63 ± 0.69^**^
1	79.69 ± 1.52^**^	13.20 ± 1.87^**^	7.09 ± 0.73

**Notes.**

All data are expressed as mean ±  sd ( *n* = 3).

* *P* < 0.05.

** *P* < 0.01 vs. control. HA, 300 kDa, 1 mg/mL.

The cell cycle stages 3 of hAECs were tested after 48 h of HA addition.

### Effects of HA on senescence of hAECs

**Figure 5:**
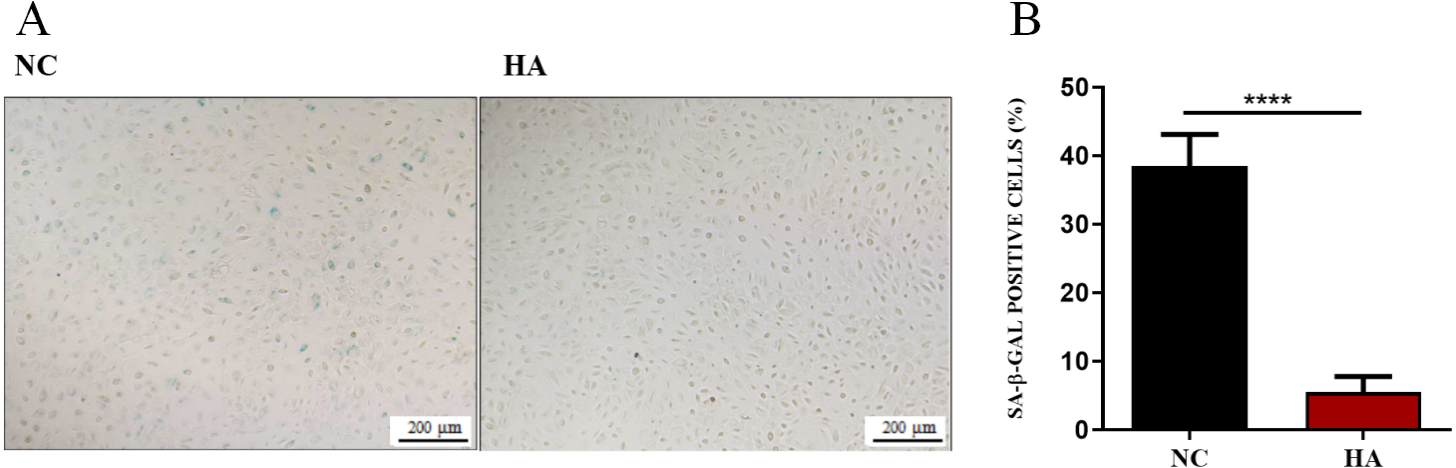
Effect of 300 kDa HA on senescence of hAECs. (A) Representive bright field images of effects of HA on hAEC senescence. (B) Percentage of SA-β-Gal positive cells with or without HA treatment (*n* = 9). ^∗∗∗∗^*P* < 0.0001.

We then determined whether HA could ameliorate the senescnece of hAECs. After 48 h of HA treatment, compared with the control, the percentage of SA- β-Gal positive cells significantly decreased from 38.48 ± 1.56% to 5.51 ± 0.77%, suggesting the significant effect of 300 kDa HA on retarding the senescence of hAECs (Fig. 5).

### Acting mechanism of HA promoting the proliferation of hAECs

**Figure 6:**
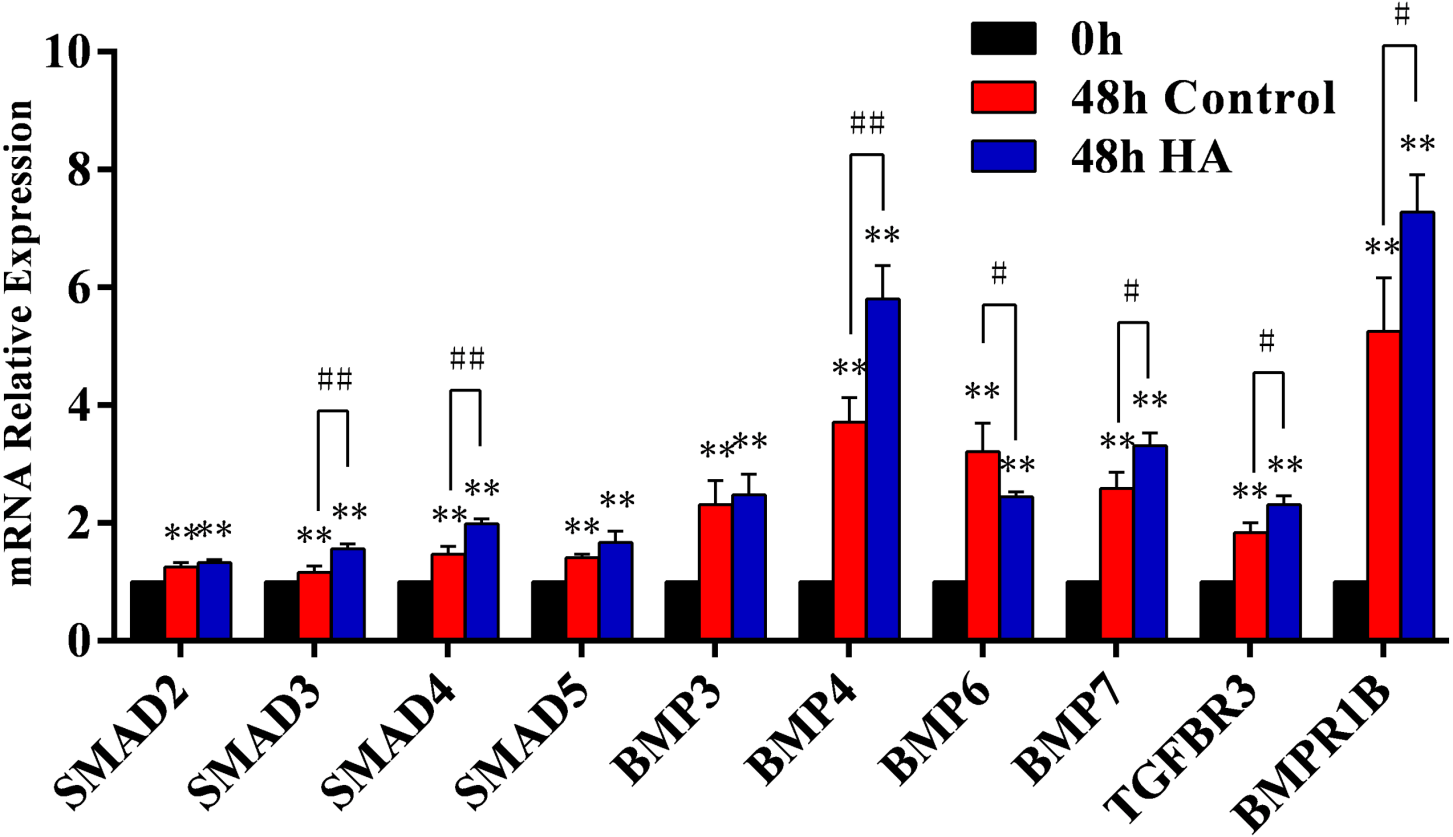
Effect of 300 kDa HA on relative expression of TGF-β/BMP signaling pathway genes in hAECs. ^∗^*P* < 0.05, ^∗∗^*P* < 0.01 vs. 0 h control group; ^#^*P* < 0.05, ^##^*P* < 0.01 vs. 48 h control group. All data are expressed as mean ± sd (*n* = 3). HA, 300 kDa, 1 mg/mL. Gene expression was tested after 48 h of HA addition.

We further investigated the signaling pathway involved in the HA-induced proliferation. In our preliminary screening by PCR microarray, the obvious fold change of signaling pathway genes (*SMAD5*, *BMP3*, *BMP4*, *BMP6*, *BMP7*, *TGFBR3*, and *BMPR1B*) in hAECs was observed after HA treatment (Table 3). Further validation by RT-qPCR demonstrated that HA significantly up-regulated the expression of *SMAD3*, *SMAD4*, *BMP4*, *BMP7*, *TGFBR3*, and *BMPR1B* (*P* <  0.05 or *P* <  0.01), but significantly down-regulated the expression of *BMP6* (*P* <  0.05) (Fig. 6). These results strongly suggest the correlation between the pro-proliferative effect and the TGF-β/BMP signaling pathway.

**Table 3:** Effect of 300 kDa HA on relative expression of TGF-β/BMP signaling pathway genes in hAECs by PCR microarray.

TGF-β/BMP signaling pathway	Time (h)	
gene	18	36
*SMAD2*	1.05	1.03
*SMAD3*	−1.06	1.29
*SMAD4*	1.03	1.14
*SMAD5*	1.59	1.32
*BMP3*	2.49	−2.22
*BMP4*	−2.19	1.34
*BMP6*	−5.02	−11.03
*BMP7*	−4.29	4.82
*TGFBR3*	1.67	2.15
*BMPR1B*	−2.95	−1.99

**Notes.**

HA (300 kDa, 1 mg/mL) group vs. control group. The treatment time was 18 or 36 h.

**Figure 7:**
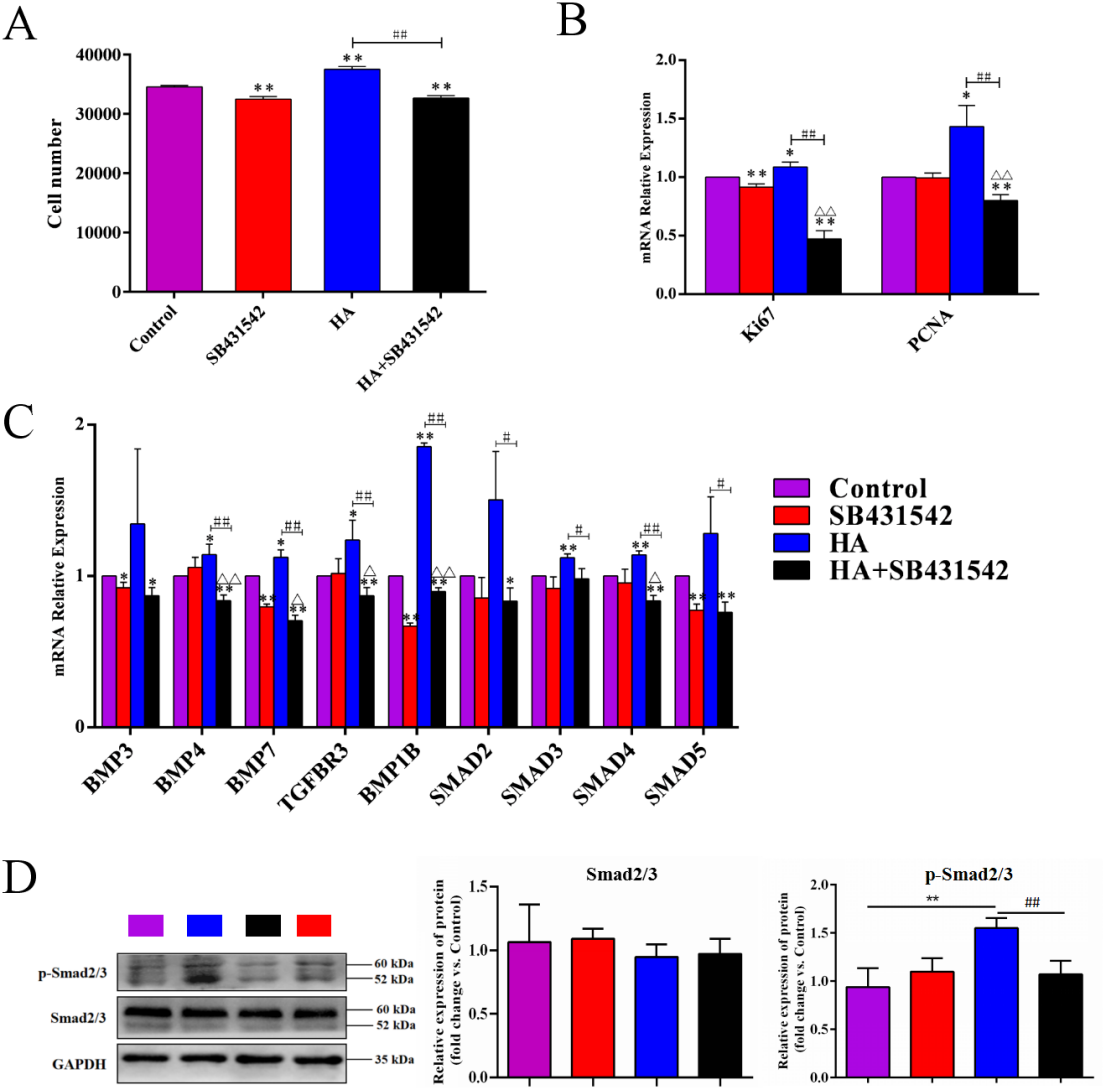
Effect of SB431542 on the cell proliferation and the TGF-β/BMP signaling pathway in hAECs. (A) Effect of SB431542 on cell proliferation of hAECs with or without HA. (B) Effect of SB431542 on relative expression of proliferation-related genes (*Ki67* and *PCNA*) with or without HA. (C) Effect of SB431542 on relative expression of TGF-β/BMP signaling pathway genes with or without HA. (D) Effect of SB431542 on relative expression of p-Smad2/3 with or without HA. ^∗^*P* < 0.05, ^∗∗^*P* < 0.01 vs. control; ^#^*P* < 0.05, ^##^*P* < 0.01 vs. HA group; Δ*P* < 0.05, ΔΔ*P* < 0.01 vs. SB431542 group. All data are expressed as mean ± sd (*n* = 3). HA, 300 kDa, 1 mg/mL. SB431542, 10 µM. Cell proliferation and gene expression were tested after 48 h of the addition of HA, SB431542, or HA+SB431542.

We further used SB431542, a specific blocker of TGF-β/BMP signaling pathway, to treat hAECs with or without 300 kDa HA exposure. As shown in Fig. 7A, the cell number in the HA group was significantly reduced after addition of SB431542 (*P* <  0.01). Consistently, the transcriptional levels of proliferation-associated factors *Ki67* and *PCNA* were significantly inhibited after SB431542 treatment (*P* <  0.01) (Fig. 7B). SB431542 could also significantly suppress the expression of TGF-β/BMP signaling genes, including *BMP4*, *BMP7*, *TGFBR3*, *BMPR1B*, *SMAD3*, *SMAD4* and *SMAD5* (*P* <  0.05 or *P* <  0.01) (Fig. 7C). Furthermore, HA treatement could significantly incease the expression of phosphorylated Smad2/3 (p-Smad2/3), while this effect could be reversed by the addition of SB431542 (Fig. 7D). These results strongly suggest that the pro-proliferative effect of 300 kDa HA on hAECs is associated with the TGF-β/BMP signaling pathway.

## Discussion

hAECs are an emerging stem cell source with great application potentials in the field of regenerative medicine (Insausti et al., 2014; Miki, 2018). However, the limited proliferative capacity of hAECs has severely hindered their in vitro expansion and further application. Therefore, searching for suitable pro-proliferative strategies for hAECs is of great practical significance. Currently, there are two main strategies to promote the proliferation of hAECs, i.e., gene modification of the cells and new medium formulation (Zhou et al., 2013; Fatimah et al., 2012). Considering the safety issues that are still not completely addressed for the strategy of gene modification, new medium formulation is accepted as a more promising strategy. One of the examples is supplementing EGF into culture medium of hAECs as described above (Fatimah et al., 2012). In the present study, supplementation of 300 kDa HA (1 mg/mL) significantly enhanced the proliferation of hAECs and did not affect their phenotypic characteristics. The same as EGF, 300 kDa HA increased the cell proportion in S and G2/M phases (Fatimah et al., 2012). Nonetheless, superior to EGF addition, 300 kDa HA treatment increased the stemness gene/protein expression and anti-inflammatory cytokine secretion, and well maintained the multilineage differentiation capability of hAECs (Fatimah et al., 2012). It should be mentioned that one way to harvest more cells would be to increase the doubling rate, but another possible way is to increase the proliferative lifespan. Thus, in this study, we also tested the effect of HA on the senescence of hAECs and found a significant senescence delaying effect. It is supported by the fact that accumulation of diploid (G0/G1) phase cells can also be a result of cells entering replicative senescence, and thus an increased percentage of cells passing through the G1/S phase check point may be an indicative of reduction in early cellular senescence. These results collectively indicate that HA could increase the cell production by enhancing both the proliferating rate and the proliferating lifespan of hAECs. Admittedly, however, in the experiment studying the cell cycle progression, the population of hAECs used was not synchronized in this study. It is a more rigorous method for studying cell cycle progression to synchronize cells using a double thymidine block (for example) and then release it to study the timely progression of the synchronized cells through the entire cell cycle (Chen & Deng, 2018). In the future, we will perform this study with synchronized cells to give more accurate data of timely progression and percentage of cells present at each phase of the cell cycle progression.

As a ubiquitous component of cellular content in vertebrates, HA has exhibited a wide range of regulatory abilities on cell physiology. These regulatory abilities have been proved to be highly dependent on the molecular weight of HA (Cyphert, Trempus & Garantziotis, 2015; Zhao et al., 2018; Coulson-Thomas et al., 2014; Gesteira et al., 2017). In terms of cell proliferation, it is generally believed that high molecular weight HA (HMWHA) inhibits, while low molecular weight HA (LMWHA) promotes cell proliferation (Cyphert, Trempus & Garantziotis, 2015). Normally, 1,000 kDa is considered to be the cut-off to distinguish the two kinds of HA. For example, under in vitro culture conditions, 351∼600 kDa HA (Part# HA500K-1, Lifecore Biomedical) promotes the proliferation of human lymph node lymphatic endothelial cells (hLLECs) (Sun et al., 2019); 500–1,000 kDa HA (Adant^®^) promotes the proliferation of human adipose-derived stem cells (hADSCs) (Moreno et al., 2015); 1,000 kDa HA inhibits the proliferation of human chorionic villi-derived mesenchymal stem cells (hCVMSCs) (Liu et al., 2008); >3,000 kDa HA isolated from human amnion inhibits the proliferation of inflammatory cells (Tseng, 2016). Consistent with these results, in the present study, 300 kDa HA promotes, and 1,000 kDa HA inhibits the proliferation of hAECs. However, aside from the molecular weight, the cell type is another influence factor for the effect of HA on cell proliferation. For example, 340 kDa HA inhibits the proliferation of human pulmonary vascular smooth muscle cells (Papakonstantinou et al., 1998); >1,800 kDa HA (Part# HA2M-5, Lifecore Biomedical) promotes the proliferation of hLLECs (Sun et al., 2019). In the present study, 50 kDa HA inhibits the proliferation of hAECs. These cell-specific effects may originate from the special properties of the cells. As for hAECs, we preliminarily speculate that it may be linked to the lack of telomerase, low expression of surface receptor CD44, and a more than doubled cell cycle duration (∼57 h for hAECs estimated by DT in Fig. 1) compared to other adult stem cells (∼23 h) (Figs. 1 and 2) (Liu et al., 2016; Orford & Scadden, 2008).

**Figure 8:**
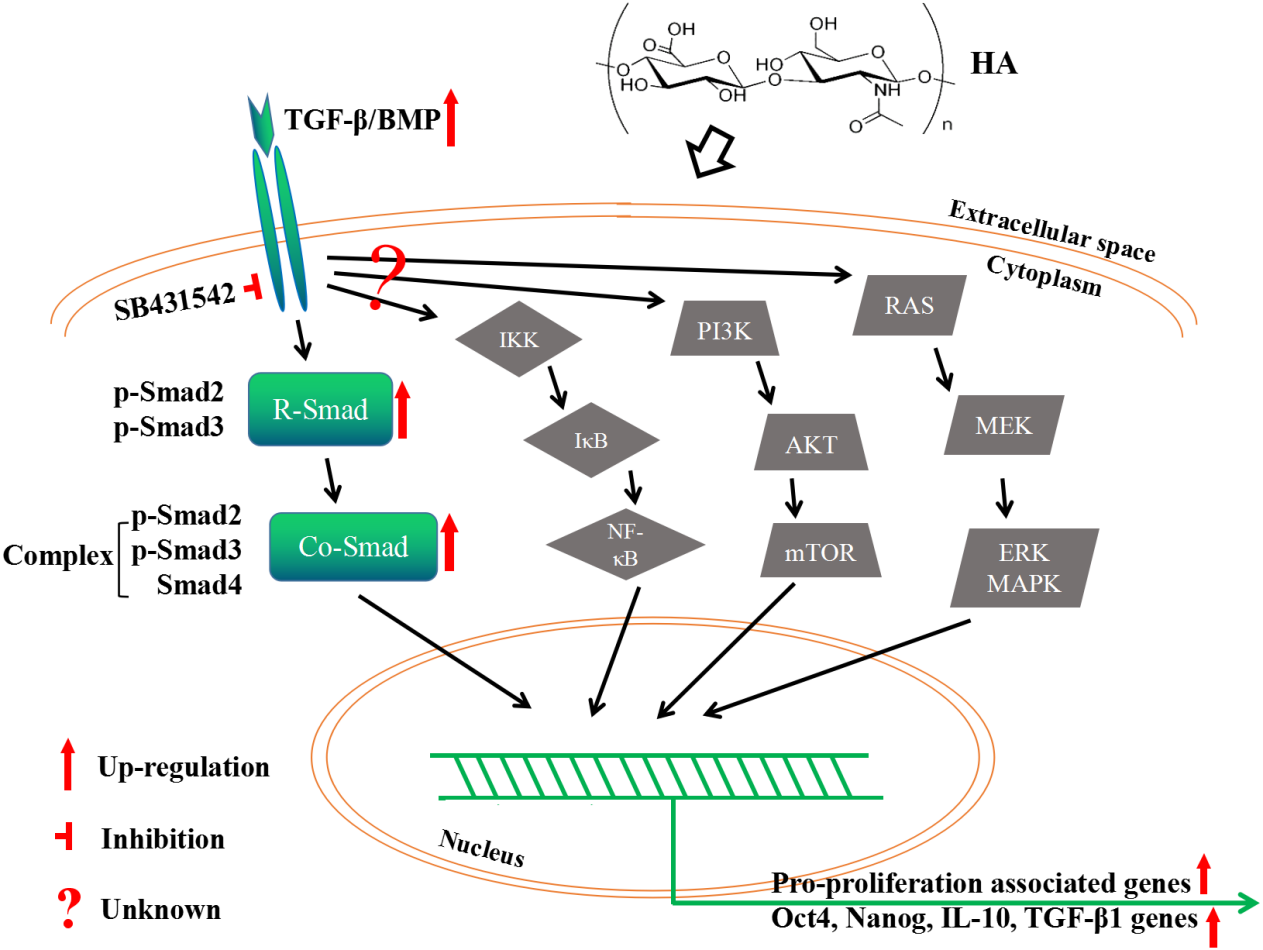
A proposed mechanism by which HA ameliorates the proliferative ability of hAECs.

As the primary cell surface receptor for HA, CD44 plays a critical role in signaling transduction in HA-mediated cell physiology (Dicker et al., 2014; Cyphert, Trempus & Garantziotis, 2015; Kota et al., 2014). The different effects of HMWHA and LMWHA on the cell proliferation can be mainly attributed to the different binding potentials of the two kinds of HA to CD44, which has been well documented in many reports (Riehl et al., 2015; Murakami et al., 2019; Song et al., 2019; Kothapalli et al., 2008; Lokeshwar, Iida & Bourguignon, 1996; Sun et al., 2019; Liu et al., 2016; Bourguignon et al., 2013; Khaing et al., 2011; Mo et al., 2011; D’agostino et al., 2017). One possible explanation of this effect is that different kinds of HA possess diverse abilities to cluster the membrane receptors (Cyphert, Trempus & Garantziotis, 2015). Namely, HMWHA increases membrane receptor clustering strength, while LMWHA shows no apparent effect on or even decreases the membrane receptor clustering strength (Cyphert, Trempus & Garantziotis, 2015). In this theory, the clustering strength is also influenced by cell type, which can be used to explain the effect of HA on some particular cell types, maybe including hAECs used in the present study (Cyphert, Trempus & Garantziotis, 2015). In spite of this, the interaction between HA and these receptors may initiate the cell signaling by distinct pathways (Cyphert, Trempus & Garantziotis, 2015; Riehl et al., 2015; Murakami et al., 2019; Song et al., 2019; Kothapalli et al., 2008; Sun et al., 2019; Liu et al., 2008; Bourguignon et al., 2013; Khaing et al., 2011; Mo et al., 2011; D’agostino et al., 2017). This is supported by a number of reported observations. Besides the examples aforementioned in the Introduction section, 750 kDa HA promotes the proliferation of mouse small intestinal epithelial stem cells through CD44- and TLR4-dependent signaling (Riehl et al., 2015), 20–500 kDa HA and >  1,000 kDa HA promote the proliferation of human vascular smooth muscle cells (hVSMCs) via CD44-ERK-Cyclin D1 axis and CD44-Rac-Cyclin D1 axis, respectively (Kothapalli et al., 2008). In the present study, the pro-proliferative effect of HA on hAECs is mediated by the canonical TGF-β/BMP signaling pathway. It is known that TGF-β/BMP signaling is mediated through Smad (canonical) and non-Smad (non-canonical) pathways. Canonical TGF-β/BMP signaling activates Smad proteins, while the non-canonical one activates MAPK, NF-κB or PI3K/AKT signaling (Weiss & Attisano, 2013). We have shown that HA treatment modulates the RNA expression of several TGF-β superfamily regulatory proteins, and the expression of p-Smad2/3 proteins. However, a CONTRADICTION WAS FOUND IN OUR RESULTS. THAT IS, ALTHOUGH increased expression of the R-Smad p-Smad2/3 and co-Smad Smad4 would seem to enhance TGF-β/Smad signaling, but increased expression of *TGFBR3*, commonly considered to be a competitive inhibitor of canonical TGF-β signaling, might seem to counteract this effect. If this is true, the expression of *TGFBR3* should be regressed to achieve higher cell proliferation rate in future exploration. Nonetheless, there is another possibility that upregulation of *TGFBR3* and its corresponding protein promotes the proliferation by activation of non-canonical TGF-β/BMP signaling. Therefore, it is worthwhile to investigate whether the PI3K/AKT or other non-canonical pathways are also implicated here. Overall, we may propose that: addition of HA activates TGF-β/BMP signaling ligands; the signaling then transduct along (at least partially) canonical TGF-β/BMP signaling to phosphorylate R-Smad Smad2/3; phosphorylated R-Smads form a complex with Co-Smad Smad4; the Smad complex is imported into the nucleus and regulates the expression of target genes by direct binding to the target gene promoter and/or through the interaction with transcriptional cofactors in a cell type-specific manner; meanwhile, the role of non-canonical TGF-β/BMP signaling here is still unknown yet (Fig. 8).

However, it should be pointed out that although a minority of the hAECs population (17.6%) are positive for CD44 (a low CD44 expression level), the fact that CD44 is a known receptor for HA should merit further investigations. It seems plausible that HA may be signaling a proliferative stimulus to a subset of hAECs that are CD44-positive. It would be very interesting to see whether the CD44-positive population of hAECs were represented in the cells that demonstrated enhanced proliferation upon stimulation with HA. One could envision FACS sorting hAECs into CD44-positive and negative populations and seeing whether the effects of HA on proliferation are different between these populations. This would help tease out the mechanism of action of the phenomenon. Alternatively, similarly, it is worth investigating whether the interaction between HA and another known HA receptor RHAMM (CD168) potentially exsisting in hAECs is a relevant mechanism of the different phenomena. However, it is still unknown yet whether hAECs express CD168 to the best of our knowledge, which will be an upcoming work for us.

It is also noteworthy the occurrence of epithelial-mesenchymal transition (EMT), another important biological process coupling with the in vitro proliferation process of hAECs, as documented in several literatures (Richardson & Menon, 2018, Richardson et al., 2019; Pratama et al., 2011; Stadler et al., 2008; Janzen et al., 2016). EMT allows epithelial cells to lose the polarization, to exhibit mesenchymal cell phenotype, and to gain a more active capacity on migration, invasiveness, and ECM secretion (Kalluri & Weinberg, 2009). It has been reported that increase of endogenous or exogenous HA could stimulate cellular EMT and proliferation (Papakonstantinou et al., 1998; Song et al., 2019). These reports also revealed the critical role of HA/its receptors (e.g., CD44 and RHAMM) interaction and TGF-β signaling in the process of EMT (Zoltan-Jones et al., 2003; Song et al., 2019; Roy et al., 2015; Porsch et al., 2013). hAECs can produce TGF-β in an autocrine fashion, and TGF-β itself is one of the major inducers of EMT (Alcaraz et al., 2013). In the present study, the involvement of TGF-β signaling suggests possible occurrence of EMT during proliferation of hAECs. As a kind of vital physiological process, EMT may be beneficial to tissue repair after the transplatation of hAECs, and may later restore the cells into the epithelial status by the process of mesenchymal-epithelial transition (MET) (Roy et al., 2015; Richardson & Menon, 2018; Pratama et al., 2011). It has been reported that during the proliferation of hAECs, the self-renewal and differentiation potential of hAECs and their cellular functions typically decrease (Pratama et al., 2011; Stadler et al., 2008; Roy et al., 2015; Richardson & Menon, 2018). However, in the present study, HA could remarkably promote the production of the stem cell pluripotent factors Nanog and Oct-4, as well as the immunosuppressive factors IL-10 and TGF-β1 in hAECs, suggesting that HA could ameliorate the stemness and cellular functions of hAECs.

In addition, as a special type of perinatal stem cells, hAECs were found to possess the charicteristics of both ESCs and MSCs, which implicates their multi-functional potentials. hAECs express ESC markers such as SSEA3, SSEA4, TRA1-60, TRA1-81, and also MSC markers such as CD73, CD90, CD105 (Miki, 2018; Roy et al., 2015). In the present study, we verified the same phenomenon that hAECs expressed CD73. The MSC-like phenotype of hAECs is also reflected by the MSC-like immunomodulatory properteis. hAECs express HLA-G molecules, which has been reported to modulate the immune response of T-regulatory cells to induce tolerance to cell or tissue grafts (Strom & Gramignoli, 2016). At the same time, hAECs display quite low immunogenicity, owing to their low (but not negative) expression of HLA-A, -B, -C and -DR surface antigens that induce graft recognition (Strom & Gramignoli, 2016). However, because HLA-A, -B, -C and -DR surface antigens are not completely negatively expressed, the possible biosafety issues that may caused by immuno-rejection should be concerned and carefully evaluated before future applications (Yong et al., 2018; Herberts, Kwa & Hermsen, 2011). Regarding biosafety, the merits for hAECs are their high genetic stability and low tumorigenicity due to their unmodified genes and missing telomerase genes (Miki, 2018; Yong et al., 2018; Herberts, Kwa & Hermsen, 2011). Therefore, the application of hAECs is promising, but systematic safety assessment would be nesseary before practical applications.

## Conclusions

In summary, this work demonstrates that 300 kDa HA (1 mg/mL) was able to ameliorate the proliferative ability of hAECs without changing their characteristic phenotypes and differentiation ability through the activation of TGF-βBMP signaling. The proportion of hAECs transits from G0/G1 phase to S and G2/M phases was significantly elevated, and the population doubling time of hAECs was significantly shortened. Futhermore, HA could effectively increase the expression of stem cell pluripotent factors and the secretion of anti-inflammatory cytokines of hAECs. Although the pro-proliferative efficacy still needs improving, our strategy provides a possible route for in vitro proliferation of hAECs, which could facilitate the future application of hAECs in regenerative medicine.

##  Supplemental Information

10.7717/peerj.10104/supp-1 Supplemental Information 1 Supplemental Files

10.7717/peerj.10104/supp-2 Supplemental Information 2 Miame Checklist

## Additional Information and Declarations

### Microarray Data Deposition

The following information was supplied regarding the deposition of microarray data: The microarray dataset is available at GEO: GSE157002. This data is also available at Figshare: Xiao, Jian-Hui (2020): pcr microarray data. figshare. Dataset. https://doi.org/10.6084/m9.figshare.11653278.v1.

### Data Availability

The following information was supplied regarding data availability: The data is available at Figshare: Xiao, Jian-Hui (2020): Hyaluronic acid ameliorates the proliferative ability of human amniotic epithelial cells through activation of TGF-β/BMP signaling. figshare. Dataset. https://doi.org/10.6084/m9.figshare.11653212.v1.
