# Supplementary material for: Hyaluronic acid ameliorates the proliferative ability of human amniotic epithelial cells through activation of TGF-β/BMP signaling
Source: PeerJ. 2020 Sep 30;8:e10104. doi: 10.7717/peerj.10104 (PMC7532780; doi:10.7717/peerj.10104)
Supplement: Supplemental Information 1 [file peerj-08-10104-s001.docx]

**Hyaluronic acid promotes the proliferation of human amniotic epithelial cells via TGF-β/BMP signaling pathway**

Ya-Bing Tian^1†^, Nuo-Xin Wang^1, 2†*^, Chang-Yin Yu^3*^, Ru-Ming Liu^1, 2^, Yi Luo^1, 2^, Jian-Hui Xiao^1, 2*^

^1^Zunyi Municipal Key Laboratory of Medicinal Biotechnology, ^2^Center for Translational Medicine, ^3^Department of Neurology, Affiliated Hospital of Zunyi Medical University, Zunyi 563003, China

**Supplementary figures**


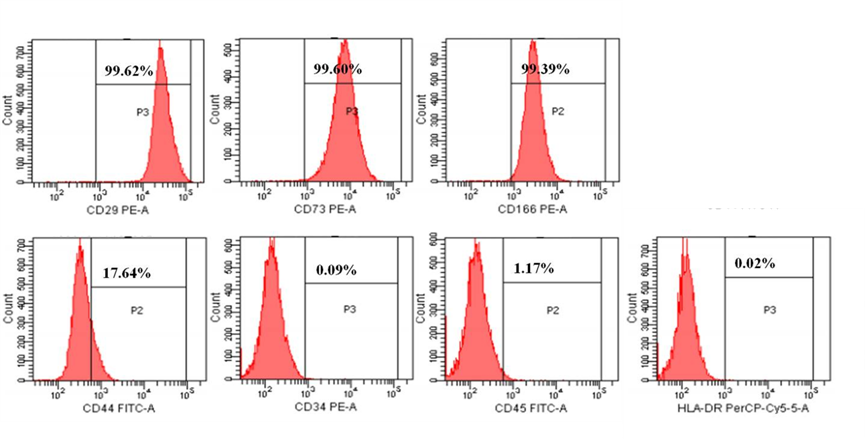


**Figure S1. Phenotypic analysis of hAECs by flow cytometry.**


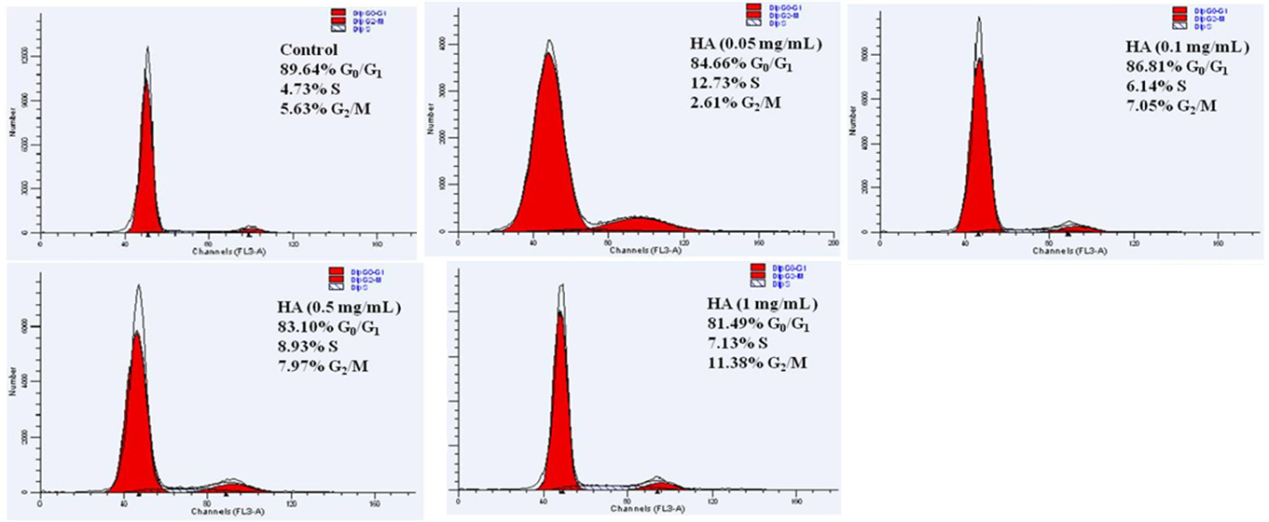


**Figure S2. Cell cycle analysis of hAECs by flow cytometry.**
