# Supplementary material for: Hyaluronic acid ameliorates the proliferative ability of human amniotic epithelial cells through activation of TGF-β/BMP signaling
Source: PeerJ. 2020 Sep 30;8:e10104. doi: 10.7717/peerj.10104 (PMC7532780; doi:10.7717/peerj.10104)
Supplement: Supplemental Information 2 [file peerj-08-10104-s002.docx]

Miame Checklist

(Sections in BOLD represent what each researcher must record about every slide and/or experiment)

**Our response is behind #**

Part 1 Experiment description

**- mouse type #human**

**- experimental variables (runners vs. non-runners, high fat vs. low fat) #hAECs at 0 hour vs. hAECs at 18 hour vs. hAECs at 36 hour**

**- n-count #6 samples (hAECs with or without HA treatment at three different time points)**

**- tissues used for slide #hAECs**

**- mouse age, and other variables (wean weight, pooled samples, etc.) #Unrelated**

Part 2 Array design.

**- Array series #PAHS-035Z(TGF-BMP), PAHS-039Z（JAK-STAT）,PAHS-043Z（Wnt）,PAHS-058Z（PI3K-AKT）,PAHS-059Z(Notch）,PAHS-061Z（MAPK）,PAHS-078Z（Hedgehog）**

**- Deconvoluted spot list with gene names**

**#https://figshare.com/articles/Hyaluronic_acid_ameliorates_the_proliferative_ability_of_human_amniotic_epithelial_cells_through_activation_of_TGF-_BMP_signaling/11653212**

**- Array type (mouse, human, cDNA, oligo, number of genes) # human, cDNA, 384 well plates**

**- Array size #384 well plates**

**- Slide type (and coating) #gene specific primers-anchored 384 well plates**

Part 3 Samples

“The MIAME 'sample' concept represents the biological material (or biomaterial) for which the gene expression profile is being established. This section is divided into three parts which describe the source of the original sample (such as organism taxonomy and cell type) and any biological in vivo or in vitro treatments applied, the technical extraction of the nucleic acids, and their subsequent labeling.”

**- Cy3/Cy5 labels for tissues #Unrelated**

**- Dye swap? Or reference control? # Reference control**

**- Labelling protocol used # SYBR Green labeling according to the Real time PCR Kit**

**- Sample extraction protocol used**

**#https://figshare.com/articles/Hyaluronic_acid_ameliorates_the_proliferative_ability_of_human_amniotic_epithelial_cells_through_activation_of_TGF-_BMP_signaling/11653212**

**- Amount of sample labelled #All samples**

Part 4 Hybridizations

“This section defines the laboratory conditions under which the hybridizations were carried out. Other than a free-text description of the hybridization protocol, MIAME requires that a number of critical hybridization parameters are explicitly specified: choice of hybridization solution (such as salt and detergent concentrations), nature of the blocking agent, wash procedure, quantity of labeled target used, hybridization time, volume, temperature and descriptions of the hybridization instruments.”

**- Hybridization protocol #**

**https://figshare.com/articles/Hyaluronic_acid_ameliorates_the_proliferative_ability_of_human_amniotic_epithelial_cells_through_activation_of_TGF-_BMP_signaling/11653212**

**- ALL modifications and deviations from the protocol # see hybridization protocol**

**- Manual hybridization or automatic chamber? #automatic chamber**

**- Number of slides done at the same time # 1 slide**

**- Hyb time # see hybridization protocol**

**- Number of washes # see hybridization protocol**

**- Amount of labelled sample hybridized #All samples**

**- Labelling efficiency #almost 100%，SYBR Green have an almost 100% labelling efficiency on double stranded DNA.**

Part 5 Measurements

“Image data should be provided as raw scanner image files (such as TIFF), accompanied by scanning information that includes relevant scan parameters and laboratory protocols.”

**- Which version of scanner software used #Applied Biosystems7500**

**- Laser power for scan #Default laser power of the instrument**

**-** **Instrument model numbers #Applied Biosystems7500**

**- Must save original .tiff format images (composite image is optional) #Unrelated**

For each experimental image, a microarray quantification matrix contains the complete image analysis output as directly generated by the image analysis software (normally provided as separate spreadsheet-type files). Note that for a given image this is a 2D matrix, where array elements (spots or features) constitute one dimension and quantification types (such as mean and median intensity, mean or median background intensity) are the second dimension.

**- Normalization protocol #RMA**

**- Does the scanner software subtract background? How much? #Default parameters of the instrument**

**- Spot raw values, background intensity, ch1 and 2 intensity, etc. #Default parameters of the instrumen**

**- Corresponding gene name #Default parameters of the instrument**

**- Methods of analysis (MAN, Spotfire, Genespring) be detailed. #Default parameters of the instrument**

**- Normalized to controls? Controls removed? All normalization parameters #Default parameters of the instrument**

**- Name of Images, Experiment, and location of files.**

**#https://figshare.com/articles/Hyaluronic_acid_ameliorates_the_proliferative_ability_of_human_amniotic_epithelial_cells_through_activation_of_TGF-_BMP_signaling/11653212**

**- Lowess or other normalization if used (and parameters) #Default parameters of the instrument**

Finally, the gene expression matrix (summarized information) consists of sets of gene expression levels for each sample. If microarray quantification matrices can be considered spot/image centric, then the gene expression matrix is gene/sample centric. At this point, the expression values may have been normalized, consolidated and transformed in any number of ways by the submitter in order to present the data in a form amenable to scientific analysis. Rather than attempting to impose a standard for gene expression values, MIAME indicates preferred detailed specifications of all numerical calculations applied to unprocessed quantifications in (b) that have led to the data in (c). Experimenters are encouraged, though not required, to provide reliability indicators (such as s.d.) for each data point.

**- Output file #Unrelated**

**- Normalized ratios #Unrelated**

**- Numerical manipulations #Unrelated**

**- Cut off values #Default parameters of the instrument**

Part 6 Normalization controls

“A typical microarray experiment involves a number of hybridization assays in which the data from multiple samples are analyzed to identify relative changes in expression levels, identify differentially expressed genes and, in many cases, discover classes of genes or samples having similar patterns of expression.”

**- Hypothesis #**2^-ΔΔCt^

**- Gene expression patterns found #Unrelated**

**- Controls used, normalization methods used (see above) #RMA under Default parameters of the instrument**

**Controls used see**

**https://figshare.com/articles/Hyaluronic_acid_ameliorates_the_proliferative_ability_of_human_amniotic_epithelial_cells_through_activation_of_TGF-_BMP_signaling/11653212**
